# Feasibility, Acceptability, and Initial Efficacy of a Digital Intervention to Improve Consumption of Foods Received within a National Nutrition Assistance Program

**DOI:** 10.3390/nu15020438

**Published:** 2023-01-14

**Authors:** Melissa C. Kay, Nour M. Hammad, Tracy Truong, Sharon J. Herring, Gary G. Bennett

**Affiliations:** 1 Department of Pediatrics, Duke University, Durham, NC 27708, USA; 2 Department of Nutrition, Harvard T.H. Chan School of Public Health, Boston, MA 02115, USA; 3 Department of Biostatistics and Bioinformatics, Duke University, Durham, NC 27708, USA; 4 Program for Maternal Health Equity, Center for Urban Bioethics, Temple University, Philadelphia, PA 19140, USA; 5 Department of Psychology and Neuroscience, Duke University, Durham, NC 27708, USA

**Keywords:** diet quality, digital, WIC, maternal diet, feasibility

## Abstract

Many mothers are vulnerable to poor diet quality, particularly those living in low-income households. The Special Supplemental Nutrition Program for Women, Infants, and Children (WIC) provides nutrient-rich foods through its benefits packages, but many WIC participants are not redeeming them. We assessed the feasibility and acceptability of a digital intervention to support redemption and consumption of WIC-approved foods to ultimately improve diet quality. We enrolled 54 maternal–child dyads receiving WIC benefits to receive three to four weekly text messages for 12 weeks focused on behavioral goals to improve consumption of WIC-approved foods. We assessed engagement with weekly tracking messages and satisfaction and collected 24 h dietary recalls to assess preliminary efficacy on dietary intake. Participants were mostly non-Hispanic white (63%) and working (63%), and responded to 7.4 (standard deviation: 4.6) of the 12 weekly messages. Half (n = 27) were high engagers (responded to 80% or more of weekly messages), with 28% (n = 15) responding to all messages. Most felt the feedback (94%) and tips (87%) were helpful and would recommend the program (91%). More were consuming leafy green vegetables compared to baseline (*p* = 0.01). Mothers of children enrolled in WIC found a text messaging intervention focused on consumption of WIC-approved foods enjoyable and helpful.

## 1. Introduction

The first two years of life are a critical time for obesity prevention since that is when dietary patterns emerge that serve as the foundation for future eating patterns, which can have negative consequences for diet quality and obesity risk [1,2,3]. Children consuming diets higher in energy-dense foods (i.e., French fries, desserts, sugar-sweetened beverages) during infancy have an increased risk of being overweight later in childhood [4,5]. Parents and caregivers play a key role in structuring early experiences with food and eating [6]. As infants wean from a milk-based diet, they learn what, when, and how much to eat from those around them, particularly their mother [7]. Thus, mothers play an important role in shaping young children’s dietary habits and are critical in preventing childhood obesity [8,9]. However, for many mothers, diet quality is suboptimal [10,11], particularly among those living in lower-income households [12]. Few interventions aim to improve maternal diet quality as a way to shape infant diet [13].

The Special Supplemental Nutrition Program for Women, Infants, and Children (WIC) serves seven million low-income, nutritionally at-risk pregnant and postpartum women, infants, and children up to the age of 5 years [14], and is an important program for reaching and promoting optimal nutrition among low-income populations at higher risk for obesity. One of the many benefits of WIC is the monthly food packages that include vouchers for supplemental foods that provide essential nutrients for mothers and young children [15]. WIC participants are expected to redeem all their benefits to meet program goals of achieving adequate dietary intake. However, many food items are going unredeemed [16]. Among the numerous barriers to redemption [17], participants struggle with knowing how to cook and prepare some of the WIC-approved foods, leading to decreased redemption and waste [18,19].

Given the ubiquity of mobile phone use [20,21], offering a text messaging intervention to support consumption of WIC-approved foods could help improve WIC redemption rates and maternal diet quality to ultimately impact child diet. Some WIC programs have successfully used web- and mobile-based applications to deliver nutrition education [22,23]. Previously conducted text message-based interventions targeting WIC caregivers have been successful in facilitating early contact between participants and breastfeeding peer counselors, promoting obesity prevention in infants, and controlling gestational weight gain [24,25,26]. However, few use text messaging to support behavioral change, lacking specific techniques such as self-monitoring and goal tracking, and none focus on improving diet quality [27]. Conducting research to fill this gap is critical given the increased risk for poor diet quality and obesity among families living in low-income households.

In the current study, we created Healthy Roots, a behavioral intervention delivered via text message to improve the diet quality of primary caregivers of young children enrolled in WIC by increasing consumption of WIC-approved foods [28]. The advantage of Healthy Roots is that it is delivered using an interactive, automated text messaging intervention designed to support behavior change through goal setting, self-monitoring, and tailored feedback. We tested the feasibility of Healthy Roots using a prospective cohort design. Using the framework outlined by Bowen et al. [29], this paper presents the acceptability, reach, implementation, and preliminary efficacy of Healthy Roots as a strategy to improve consumption of WIC-approved foods, and thus improve maternal diet quality.

## 2. Materials and Methods

### 2.1. Study Design and Eligibility

The study design and protocol have been reported elsewhere [28]. Briefly, we recruited parents and caregivers ≥18 years old with children aged ≤2 years old attending WIC clinics at a network of federally qualified community health centers in North Carolina, which also serve pregnant women and children ages 0–5 through WIC. To be eligible for the study, parents were required to have a smartphone that could send and receive text messages and access the internet, be willing to send and receive study-related text messages, have an email address they checked regularly, have regular access to the internet, and be comfortable reading and writing in English. Eligibility was determined using an online screener administered through REDCap (Research Electronic Data Capture), a secure, web-based software platform [30]. Eligible participants were redirected to an online consent form and completed online surveys which included sociodemographic characteristics, anthropometrics, and WIC food preferences. Upon completion of the baseline surveys and prior to enrollment, participants were asked to complete two separate 24 h dietary recalls using the Automated Self-Administered 24 h Recall (ASA24) system developed by the National Cancer Institute (NCI) [31]. Upon completion of the ASA24s, parents were enrolled in the intervention and began receiving SMS text messages including a 2 min orientation video. After 12 weeks, participants were asked to complete two more ASA24s and a satisfaction survey. Participants received a gift card after completion of each ASA24 for a maximum amount of USD 55. All study protocols were in accordance with the ethical standards of Duke University and were approved by the Duke University Health Center Institutional Review Board. The study is registered with clinicaltrials.gov (NCT04098016).

### 2.2. Intervention Description

The intervention was fully automated and delivered via text messaging. It was modeled after an evidence-based digital obesity treatment program called iOTA [32,33,34,35,36,37,38,39]. Similar to iOTA, intervention components were delivered using SMS text messages, interconnected algorithms, and content libraries [40]. Intervention development was also informed by the Social Cognitive Theory and included effective behavior change techniques such as goal setting, self-monitoring, and tailored feedback [41,42].

#### Behavioral Change Goals

Each participant received a total of six goals that changed every two weeks across the 12-week intervention. Goal creation was guided by formative work [28], empirical support for improving diet quality, and ease of self-monitoring. Each goal focused on a specific category of WIC-approved foods (Table 1), and the recommended amount and frequency of consumption was guided by the 2020–2025 *Dietary Guidelines for Americans* [43]. During each 2-week goal cycle, participants received three tips per week related to that goal which included recipes and simple behaviors to support meeting the goal. At the end of each week, participants were asked to self-monitor their behavior related to their assigned goal; an SMS text message prompted them to communicate their weekly tracking data, e.g., “Over the past week, how many TIMES did you eat beans (like kidney, navy, or pinto beans, chickpeas or lentils)? Please text only a number (like 0, 1, 2).” The computer algorithm provided tailored feedback according to their response. If they did not respond, they received an automated reminder. If there was still no response, they received a reminder to track the next time and a goal-related tip. Prior to receiving the first goal message, participants received a link to a 2 min orientation video that provided an overview of the intervention including the schedule, how to respond to texts, and where to go for help.

### 2.3. Measures

#### Baseline Sociodemographic and Psychosocial Variables

Sociodemographic measures were collected at baseline using standard survey questions used in previous studies. This included age, race, ethnicity, education, marital status, employment, insurance type, and number of children in the household. Height and weight were self-reported and body mass index kg/m^2^ (BMI) was calculated for each participant. Depression was assessed using the validated 8-item Patient Health Questionnaire (PHQ-8) [44]. The scale ranges from 0–24, with a score of ≥10 indicating depression. Household food security status was assessed using the 2-item screen derived from the U.S. Department of Agriculture 18-item Household Food Security Survey [45].

### 2.4. Reach and Representativeness

We collected administrative and survey data to assess the reach among WIC participants, participant enrollment (i.e., total number of WIC participants reached to recruit sample size, time to recruit sample size, and retention at 12 weeks), and sociodemographic characteristics.

### 2.5. Implementation

Intervention engagement was used to assess implementation. Engagement was operationalized as responsiveness to self-monitoring prompts for tracking behavioral goals across the 12-week pilot. Each week, participants received an automated prompt from the intervention system to track adherence to behavioral goals, delivered via text message. Tracking was considered complete if the participant responded to the weekly text. Engagement was determined by dividing the number of days with tracking data by the total number of possible tracking days. Engagement was categorized as high or low using an established cutoff of responding to 80% or more of weekly self-monitoring texts [46,47].

### 2.6. Acceptability

We assessed acceptability with the intervention (delivery and content) using quantitative and qualitative methods. Upon study completion, participants completed a post-study satisfaction survey adapted from our previous studies to assess acceptability and perceptions about the use of digital technologies for improving consumption of WIC-approved foods, as well as overall satisfaction with the intervention. Using a 5-point Likert scale from strongly disagree to strongly agree, we asked about perceptions of all elements of the intervention, including the usefulness of feedback, the personalization of texts, the timing of the texts, and whether an individual would recommend this program to others. In addition, we conducted in-depth post-intervention interviews with a subset of study participants (n = 10) to assess participants’ views and experiences of the intervention and capture additional barriers and facilitators to participation. We used a purposive sampling strategy to obtain feedback from both high and low engagers with the intervention to capture a variety of perspectives [48]. The interview consisted of 14 open-ended questions, which were developed to ensure consistency across interviews but to allow for probing, as deemed appropriate. All interviews were audio-recorded with responses kept confidential. Participants received a USD 15 gift card for participation in the interview.

### 2.7. Preliminary Efficacy

We assessed the intervention’s impact on dietary intake of the targeted food groups and overall diet quality. Dietary intake was measured using the ASA24, an automated tool that uses the United States Department of Agriculture’s (USDA) validated multiple pass method to elicit intake throughout a given day [31]. Using an unannounced protocol, participants were asked via email to complete 24 h dietary recalls at both the beginning and end of the trial. We asked participants to complete two ASA24s (ideally 1 weekend day and 1 weekday) within a 2-week period at each time point. To assess diet quality, the ASA24 dietary intake data were used to calculate a Healthy Eating Index-2015 (HEI-2015) score. The HEI is a tool developed by the USDA and NCI to determine conformance with the *Dietary Guidelines for Americans* (DGA) [49]. The HEI-2015 consists of 13 components, 9 of which assess adequacy of the diet, including (1) Total Fruits; (2) Whole Fruits; (3) Total Vegetables; (4) Greens and Beans; (5) Whole Grains; (6) Dairy; (7) Total Protein Foods; (8) Seafood and Plant Proteins; and (9) Fatty Acids, which is a ratio of unsaturated versus saturated fatty acids. The remaining assess dietary components to limit: (10) Refined Grains; (11) Sodium; (12) Added Sugars; and (13) Saturated Fat. For all components, higher scores reflect better diet quality as moderation components are reverse-scored. The 13 components sum to yield a maximum total score of 100, with higher scores reflecting greater compliance with recommendations from the 2015–2020 DGA.

### 2.8. Analysis

For descriptive analyses, variables are summarized and reported as means and standard deviations (SD). Engagement is reported as the mean tracking rate over the course of the study. Bivariate analyses using t tests and chi-square were used to examine predictors of intervention engagement. Prior to assessing changes in dietary intake and diet quality, we treated invalid baseline and 12-week ASA24 data as missing using standard protocols for obtaining valid dietary intake (i.e., having a mean daily caloric intake <600 or >5000 (n = 13)) [50]. To describe dietary intake, we report the mean usual intake for all participants with valid data at baseline (n = 54) and at 12 weeks (n = 48). Although the protocol aimed to collect two ASA24s at each time point, given the difficulties imposed by the COVID-19 pandemic on data collection, we included participants who had at least one ASA24 at each time point. According to the Dietary Assessment Primer, one 24 h dietary recall is sufficient for estimating usual intake and changes in intake among a group [51]. Analyses were conducted using R 4.1.0 (R Core Team, 2021) [52] software and *p*-values with an alpha <0.05 were considered statistically significant.

## 3. Results

### 3.1. Reach and Representativeness

**Figure 1:**
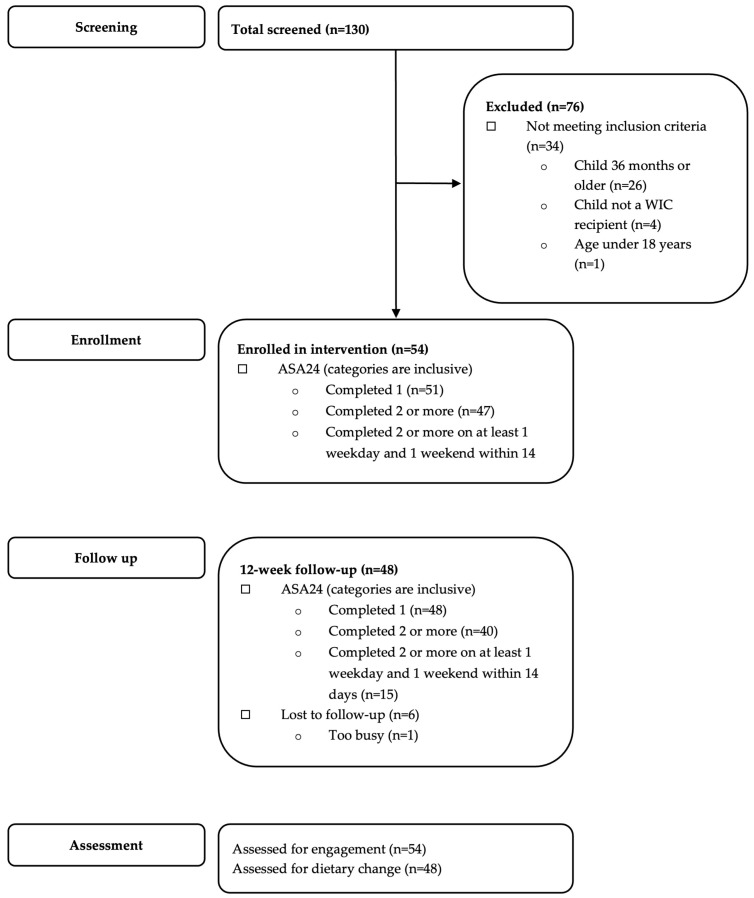
Healthy Roots Consort Diagram.

The recruitment period lasted from 3 March 2021 through 25 June 2021. The CONSORT diagram (Figure 1) shows the study flow for both recruitment and retention; 130 individuals filled out the screening survey and 34 of those individuals were ineligible; 42 declined participation or did not complete baseline activities, resulting in a 56% recruitment rate. The remaining 54 participants received the intervention. At the end of the study, 87% of participants completed the final survey (n = 47). The ASA24 surveys had different retention rates. At baseline, all participants completed at least one ASA24, 47 (87%) completed two or more and 27 (50%) completed at least one weekday recall and one weekend day (Friday, Saturday, Sunday) recall that were at most 14 days apart. At 12 weeks, 48 (89%) participants completed at least one valid ASA24, 40 (74%) completed two or more, and 15 (28%) completed at least one weekday and one weekend day recall 14 days apart.

#### Baseline Characteristics

Participants (n = 54) were all women with a mean (SD) age of 31.1 (7.7) years and a mean (SD) BMI of 33.8 (9.7) kg/m^2^ (Table 2). Most participants were white (63%), employed or looking for work (63%), not married (57%), and had obesity (65%). Nearly half (44%) were food-insecure and over a quarter had some level of depression (30%).

### 3.2. Implementation

**Figure 2:**
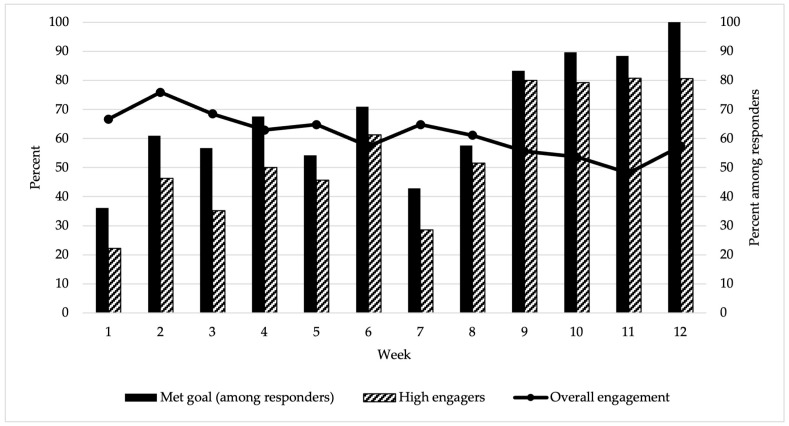
Engagement across the 12-week pilot (n = 54). The primary axis represents overall engagement across the 12-week pilot as noted by the line graph; the secondary axis represents the percent who met their goal and the percent who were high engagers (responded to 80% or more of texts) among those who responded to the texts for each week across the 12-week pilot as represented by the bar graph.

Across the 12-week intervention, participants responded on average (SD) to 7.4 (4.6) of the weekly tracking messages, equating to an overall engagement rate of 61%; 28% (n = 15) responded to all tracking messages, and 13% (n = 7) did not respond to any tracking messages. Engagement varied from a high of 76% responding to the text messages at week 2 to a low of 48% responding to the text messages at week 11 (Figure 2). As shown in Figure 2, those who were high engagers (responded to 80% or more of weekly tracking messages) were more likely to report meeting their weekly behavior change goal.

Half the sample (n = 27) were high engagers (Table 3), and half were low engagers (n = 27). Over a quarter responded to every tracking message (28%, n = 15) and 13% (n = 7) did not respond to any tracking messages. Engagement levels varied by caregiver race and ethnicity (*p* = 0.027), employment status (*p* = 0.032), and marital status (*p* = 0.023). The orientation video was viewed 53 times among 48 unique viewers. On average, viewers watched 55.5% of the 2:36 min video; the average view duration was 1:27 min.

### 3.3. Preliminary Efficacy

#### 3.3.1. Consumption of WIC-Approved Food Groups

**Figure 3:**
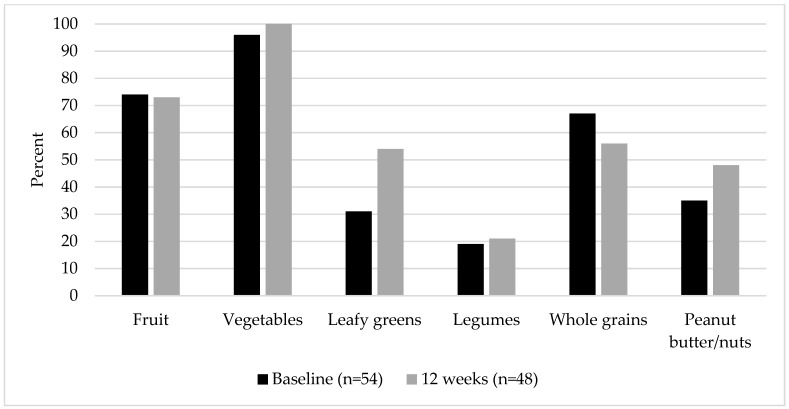
Percent of the participants enrolled in the Healthy Roots intervention consuming targeted food groups.

The percent of the sample consuming each of the targeted intervention food groups at each time point is presented in Figure 3. Compared to baseline, significantly more participants were consuming dark leafy green vegetables upon completion of the study at 12 weeks (*p* = 0.01).

#### 3.3.2. Diet Quality

As shown in Table 4, there was no significant change in HEI-2015 score between baseline 49.7 (12.4) and 12 weeks 50.5 (13.8). For individual HEI-2015 components, participants scored significantly higher at 12 weeks compared to baseline for Greens and Beans (*p* = 0.04) and Added Sugars (*p* = 0.01), and significantly lower for Sodium (*p* = 0.02). There were no significant differences in overall or component HEI-2015 scores by levels of engagement (low vs. high), adjusting for baseline scores.

#### 3.3.3. Acceptability

Regarding the frequency of texts, 85% felt the frequency or number of texts they received was just enough; 11% felt there were too many and 4% felt they did not come often enough. When asked how often participants would like to receive texts, more than half (51%) said weekly, 17% said a few times a week or none at all, 9% said daily, and 6% said monthly. Table 5 shows the intervention satisfaction questions and the proportion that responded in agreement. Most (94%) participants indicated with agreement or strong agreement that the text messages were helpful, and the goals were what they needed for choosing healthy foods for themselves and their family; many (89%) applied the skills they learned from the tips to their routine. Not all felt the text messages were personalized (74%) and only 68% said they would like to continue receiving text messages from the Healthy Roots program.

A subset of participants (n = 10) completed a post-intervention interview. The interview consisted of 14 open-ended questions based on four themes: (1) overall experience; (2) dietary change; (3) engagement; and (4) constructive feedback. When asked to characterize their overall experience in Healthy Roots, nine of the respondents reported a positive overall experience. One participant said:

“*I was able to learn how to use what WIC offered me on certain vegetables that I wouldn’t have usually bought for my family*”.

Similarly, when asked if they would recommend the program to a friend or family member, all respondents affirmed. Nine of the ten participants reported their eating habits changing after taking part in the study and that their buying habits for WIC-approved foods also changed. One participant said: “I didn’t realize I could buy so many different foods.” Seven of ten participants expressed that they were able to attempt recipes from the text messages, and most consistently responded to the text messages they received; three participants cited a reason for their non-responsiveness as being that they became busy. To gauge areas that Healthy Roots could improve, we asked participants for feedback on which aspects of the program they most disliked and other aspects that could be improved. Nine of ten respondents reported that they would have liked the program to incorporate more recipes. One participant said:

“*Finding more ways to incorporate more ideas for the harder ingredients like the greens and stuff that kids don’t like*”.

## 4. Discussion

As demonstrated by our quantitative and qualitative findings, we found that it is feasible and acceptable to use a responsive text messaging intervention with personalized, tailored feedback to support behavior change related to consumption of WIC-approved foods among primary caregivers of young children enrolled in WIC. We were successful at recruiting and retaining caregivers of young children enrolled in WIC and achieved moderate to high rates of engagement with responses to goal tracking. Most participants reported consuming more WIC-approved foods, particularly leafy green vegetables, and high satisfaction with the intervention. In fact, significant improvements in the HEI-2015 component scores for greens and beans and added sugars suggest providing parents with ideas and specific recipes can improve intake of certain foods. However, we also found decreased scores for sodium intake, which may indicate a reliance on canned foods or salty seasonings when cooking. Future programs should be more specific on adopting low-sodium techniques for preparing and cooking foods (i.e., rinsing canned vegetables and beans before using; seasoning foods with salt-free herbs and spices).

Our findings build on previous reports that WIC participants are amenable to text messaging by demonstrating their willingness to engage in text messaging for behavior change [25,53,54]; making texting a cost-effective way to reach WIC participants on a large scale and reduce burden among a hard-to-reach population [55]. This is important because WIC has experienced decreased enrollment and retention in recent years [56], and well-designed digital interventions could be used to deliver nutrition education and keep caregivers engaged and retained. The provision of nutrition education is a core tenet of the WIC program, which sets it apart from other nutrition assistance programs; WIC participants receive nutrition education at a minimum of four times a year. Traditionally, mothers and caregivers enrolled in WIC receive nutrition education during clinic visits. However, given the barriers associated with attendance at required in-person visits [57], there is a need to explore alternative education methods to deliver nutrition information and support behavior change. Our findings show caregivers were engaged and receptive to the information received via text messaging, despite the absence of costly and resource-intensive face-to-face interaction. Text messaging could also be used to fill the gap between visits and provide important nutrition education during the ever-changing period of a child’s first two years of life. High levels of satisfaction among those enrolled in Healthy Roots indicate that text messaging may be an acceptable accompaniment to WIC nutrition counseling. Offering text messaging between or in place of in-person visits could improve retention and allow time-constrained WIC staff to reach clients more effectively.

Besides meeting program requirements, offering nutrition education can increase nutrition knowledge, an important driver of consumption of healthy foods, particularly fruits and vegetables [58,59]. Increasing nutrition knowledge among caregivers enrolled in WIC has specific implications for children, as the more caregivers know about food and nutrition, the better the quality of their children’s diets [60]. Although some knowledge is important for effective behavior change [61], nutrition education alone is not always sufficient to effect dietary change [62,63]. Self-monitoring and tailored feedback are important behavior change techniques, especially for digital interventions as they can improve engagement and adherence [27,64]. For example, self-monitoring increases awareness of the food being consumed and helps elucidate barriers to positive dietary behavior changes [65]. The acceptability and preliminary efficacy of Healthy Roots are demonstrated by the level of engagement and the increased likelihood of meeting weekly goals with higher engagement, but there is room for improvement in the personalization of messages, given this was the lowest in terms of acceptability. Although engagement is critical for increasing the effectiveness of digital behavior change interventions [66], personalized text messages are more likely to be relevant, remembered, saved, and discussed with others compared to non-personalized texts [67]. However, our findings show that, overall, WIC caregivers can achieve moderate engagement in a short-term digital intervention, especially when provided tailored weekly feedback in response to self-monitoring of specific and achievable goals. These findings are important because they demonstrate the potential of a standalone text messaging intervention to engage a population at high risk for obesity and influence consumption. Although higher engagement can be achieved by combining digital technologies with human support [68,69], it is more costly and resource-intensive, and can be prohibitive to participation compared to standalone text messaging [70]. While text messaging has demonstrated effectiveness in behavior change interventions, such as weight management and smoking, few have used them to influence diet quality [27,71,72,73]. Thus, our study fills an important gap in dietary behavior change interventions.

A program like Healthy Roots has important public health implications, as it supports the actions the federal government is taking to drive solutions to challenges associated with nutrition, hunger, and health, as stated in the White House Administration’s recently released National Strategy On Hunger, Nutrition, And Health [74]. Specifically, incorporating text messaging into the WIC program would support the Administration’s goals to (1) help more families access and benefit from WIC, (2) modernize WIC, and (3) leverage WIC to improve diet quality. In Healthy Roots, all caregivers who were interviewed stated that they would recommend the program to a friend or family member. This suggests that using a text messaging intervention with recipes and tips is appealing to WIC caregivers. This could be because many parents struggle with coming up with ideas of what to cook and how to prepare foods, particularly when it comes to fruits and vegetables [53]. However, overall diet quality remained unchanged with standalone text message support for behavior change. This may be explained, partly, due to the small sample size, lack of 24 h dietary recalls on at least two days for all participants, and the short duration of the intervention. In addition, all participants received the same six goals. Future studies could benefit from a more personalized set of nutrition education and behavior change goals. Although we cannot personalize the foods participants receive as part of the WIC food package, we could tailor the text messaging intervention to focus more on the foods participants do not regularly consume but are willing to increase intake of. To better understand the effects of this intervention, and on the general WIC population, a large-scale and long-term replication of the findings is needed.

### Strengths and Limitations

A major strength of this study was the purposeful use of formative research to develop the intervention. This allows for the development of a tailored, integrated, and culturally appropriate intervention that meets the caregivers’ needs [75]. Another key strength is the automated, tailored feedback that WIC caregivers received in support of their behavior change. Follow-up protocols, including sending reminders to WIC caregivers to complete their assigned tasks, also strengthened this study. However, there are several limitations to note. The small sample size did not allow for power calculations, given the nature of the study design, i.e., a feasibility study. This is consistent with the description of a feasibility trial in Arain et al. (2010), which discusses their use for descriptive analyses rather than hypothesis testing [76]. This posed further difficulty in interpreting the results of the study and observing effects of the intervention on overall diet quality. Furthermore, all sociodemographic and psychosocial measures were self-reported and are thus subject to response bias. Lastly, WIC caregivers were asked to complete four 24 h dietary recalls through the ASA24 system, but the ASA24 system can be burdensome and has usability issues among adults with low income [77]. However, research staff provided on-demand technical support, which can help improve usability and efficiency [77]. Lastly, most WIC caregivers who participated in this study were women who were white, employed, not married, and from urban settings, which limits the generalizability of the findings.

## 5. Conclusions

A text messaging program aimed at improving consumption of WIC-approved foods to improve diet quality is feasible, well-accepted, and enjoyed by caregivers of young children enrolled in WIC. This feasibility study provides support for a larger study to assess the efficacy for improving diet quality in those most at risk for obesity by supporting redemption of WIC-approved foods. At a population level, such an intervention could have an appreciable impact by reaching caregivers through a channel they use daily. Given the ability of digital health interventions to deliver interventions remotely and to reach wide audiences, such interventions have the potential to contribute to improving the healthiness of diets.

## Figures and Tables

**Table 1:** Healthy Roots behavior change goals.

Week	WIC-Approved Food	Goal
1, 2	Fruit	Eat 2 fruits or more each day.
3, 4	Vegetables	Eat 3 vegetables or more each day.
5, 6	Legumes	Eat beans 2 times or more each week.
7, 8	100% whole wheat bread, tortillas, pasta, cereal	Eat 3 or more whole grains each day.
9, 10	Legumes	Eat nuts or peanut butter 3 times or more each week.
11, 12	Vegetables	Eat leafy green vegetables 2 times or more each week.

**Table 2:** Baseline demographic and psychosocial characteristics of parents and caregivers of young children participating in Healthy Roots (n = 54).

Characteristic		N (%) or M (SD)
Caregiver Age, years		31.1 (7.7)
Caregiver BMI, kg/m^2^		33.8 (9.7)
Relationship to child	Mother	53 (98)
	Grandparent	1 (2)
Index Child Age, months		11.6 (7.9)
Caregiver Race and Ethnicity	Non-Hispanic white	34 (63)
	Non-Hispanic Black	14 (26)
	Non-Hispanic two or more races	2 (4)
	Hispanic	2 (4)
	Did not respond	2 (4)
Education	Less than high school	3 (6)
	High school graduate	17 (32)
	Some college/vocational	16 (30)
	Associate’s degree or higher	18 (33)
Working full- or part-time/looking for work	Yes	34 (63)
	No	15 (28)
	Did not respond	5 (9)
Married	Yes	21 (39)
	No	31 (57)
	Did not respond	2 (4)
Caregiver with obesity		35 (65)
Household size		3.9 (1.2)
Number of children in household		2.1 (1.0)
Ever breastfed		40 (74)
Food-insecure		24 (44)
Depression		16 (30)

**Table 3:** Levels of engagement by sociodemographic characteristics (n = 54).

Characteristic		Engagement, n (%)	
		Low (<80%), n = 27	High (≥80%), n = 27
Caregiver Race and Ethnicity ^1,2^	Non-Hispanic White	14 (41)	20 (59)
	Non-Hispanic Black	11 (79)	3 (21)
Education	≤High school	12 (60)	8 (40)
	Some college/vocational	8 (50)	8 (50)
	Associates degree or higher	7 (39)	11 (61)
Employment ^2^	No	4 (27)	11 (73)
	Yes (full/part-time, looking)	21 (62)	13 (38)
Married ^2^	Yes	6 (29)	15 (71)
	No	20 (65)	11 (35)
Caregiver with obesity	Yes	15 (43)	20 (57)
	No	12 (63)	7 (37)
Food-insecure	Yes	12 (50)	12 (50)
	No	15 (50)	15 (50)
Depressed	Yes	20 (47)	23 (53)
	No	7 (64)	4 (36)

^1^ Excluded those who reported Hispanic (n = 2) and non-Hispanic 2 or more races (n = 2) due to small sample size. ^2^ *p* < 0.05.

**Table 4:** Changes in total and component Healthy Eating Index-2015 scores among caregivers enrolled in WIC participating in the Healthy Roots intervention.

Component	Max Points	Standard for Max Score	Standard for Min Score	Baseline (n = 54)	12-Weeks (n = 48)	Change (n = 48)
**Food and Nutrients to Increase**						
Total Fruits ^1^	5	≥0.8 cup eq per 1000 kcal	No fruit	2.0 (1.9)	1.9 (1.9)	0.0 (1.9)
Whole Fruits ^2^	5	≥0.4 cup eq per 1000 kcal	No whole fruit	2.0 (2.2)	2.1 (2.2)	0.1 (2.3)
Total Vegetables	5	≥1.1 cup eq per 1000 kcal	No vegetables	2.7 (1.7)	3.4 (1.6)	0.5 (1.8)
Greens and Beans	5	≥0.2 cup eq per 1000 kcal	No dark green vegetables or legumes	1.6 (2.0)	2.5 (2.3)	0.8 (2.4) *
Whole Grains	10	≥1.5 oz eq per 1000 kcal	No whole grains	2.9 (3.5)	3.2 (3.8)	0.4 (4.2)
Dairy ^3^	10	≥1.3 cup eq per 1000 kcal	No dairy	4.9 (3.1)	5.5 (2.9)	0.5 (3.6)
Total Protein Foods	5	≥2.5 oz eq per 1000 kcal	No protein foods	4.5 (1.0)	4.6 (0.9)	0.1 (1.0)
Seafood and Plant Proteins ^4^	5	≥0.8 oz eq per 1000 kcal	No seafood or plant proteins	2.0 (2.1)	2.3 (2.2)	0.3 (2.7)
Fatty Acids ^5^	10	(PUFAs + MUFAs)/SFAs ≥2.5	(PUFAs + MUFAs)/SFAs ≤1.2	5.2 (3.4)	4.7 (2.9)	−0.4 (4.1)
**Food and Nutrients to Limit or Decrease**						
Refined Grains	10	≤1.8 oz eq per 1000 kcal	≥4.3 oz eq per 1000 kcal	6.6 (3.3)	6.0 (3.4)	−0.9 (4.5)
Sodium	10	≤1.1 g per 1000 kcal	≥2.0 g per 1000 kcal	3.8 (2.9)	2.5 (2.6)	−1.4 (3.9) *
Added Sugars	10	≤6.5% of energy	≥26% of energy	6.3 (3.3)	7.5 (2.6)	1.3 (3.1)*
Saturated Fats	10	≤8% of energy	≥16% of energy	5.1 (3.7)	4.3 (3.0)	−0.5 (4.0)
Total Score	100			49.7 (12.4)	50.5 (13.8)	0.8 (12.9)

^1^ Includes 100% fruit juice. ^2^ Includes all forms except juice. ^3^ Includes all milk products, such as fluid milk, yogurt, cheese, and fortified soy beverages. ^4^ Includes seafood; nuts, seeds, soy products (other than beverages); and legumes (beans and peas). ^5^ Ratio of poly- and mono-unsaturated fatty acids to saturated fatty acids. * *p* < 0.05.

**Table 5:** Perceived usefulness and ease of use of intervention components among participants receiving the Healthy Roots intervention (n = 47).

Satisfaction Question	Agreement, n (%)
Overall, the feedback received on the automated text messages was helpful.	44 (94)
The text messages felt personalized.	35 (74)
The text messages were sent at a convenient time each day.	42 (89)
I found the tips easy to understand.	46 (98)
I applied the skills I learned from the tips to my routine.	42 (89)
It was easy to understand my goals.	45 (96)
I found the goals too difficult to meet.	7 (15)
The tips helped me to meet my goals.	41 (87)
I felt confident that I could follow the goals I was given.	40 (85)
My goals were what I needed to work on for choosing healthy foods for me and my family.	44 (94)
I think that I would like to continue to receive text messages from the Healthy Roots program.	32 (68)

## Data Availability

The data presented in this study are available on request from the corresponding author. The data are not publicly available due to privacy.
